# Improving performance metrics in WBANs with a dynamic next beacon interval and superframe duration scheme

**DOI:** 10.1016/j.heliyon.2024.e26468

**Published:** 2024-02-21

**Authors:** Abdulwadood Alawadhi, Abdullah Almogahed, Fathey Mohammed, Bakr Ba-Quttayyan, Adnan Hussein

**Affiliations:** a Department of Computers and Information Technology, Faculty of Engineering and Computing, University of Science and Technology, Aden, Yemen; b Department of Software Engineering, Faculty of Engineering and Information Technology, Taiz University, Taiz, Yemen; c Department of Information Technology, Faculty of Engineering and Information Technology, Taiz University, Taiz, Yemen; d Department of Computer Science, Faculty of Computer Science & Engineering, Al-Ahgaff University, Mukalla, Yemen

**Keywords:** Wireless body area networks, Superframe structure, Media access control, IEEE 802.15.4, 6G, TSK fuzzy model

## Abstract

The advancement of networking, information, and communication technologies has fueled the popularity of Wireless Body Area Networks (WBANs) in both medical (remote patient monitoring) and non-medical sectors. Due to low, medium, and high data traffic requirements, WBAN performance suffers during the synchronization process that generates periodic beacon frames between sensor nodes and the coordinator. It also suffers when sensor nodes implicitly send data to the coordinator during the fixed time slot using the Contention Access Period (CAP). In this study, we propose a solution called Dynamic Next Beacon Interval and Superframe Duration Scheme (DNBISD) to tackle these issues of the IEEE 802.15.4 standard. This standard relies on a Beacon Interval (BI) and CAP for synchronization and data transmission between sensor nodes and the coordinator. However, the standard must adapt to BI and CAP's changing traffic load requirements, resulting in drawbacks such as prolonged packet delays, increased energy consumption, and potential data loss, particularly in real-time patient monitoring applications. In order to overcome these challenges, our DNBISD scheme employs a fuzzy approach to adapt the BI and CAP based on requested synchronization and data considering input parameters like packet received ratio and buffer ratio. The inference system utilizes the Takagi, Sugeno, and Kang (TSK) fuzzy model for rational quantitative analysis. Simulations demonstrate that our proposed scheme significantly enhances data transmission, boosts the average packet delivery ratio and throughput, and reduces the coordinator's average packet loss ratio and energy consumption. Consequently, this improvement allows for more efficient data transfer among numerous nodes within the specified superframe structure.

## 1 Introduction

Several researchers predicted that 2030 would bring significant changes in technology and economic environments. In May 2019, the International Telecommunication Union (ITU) announced the International Mobile Telecommunications (IMT) 2030 standard based on wireless communication networks of the sixth generation (6G) [1], [2]. The sixth generation's (6G) objectives were to create a novel user experience and a fresh set of sensory data and experiences. The 6G wireless network, now being investigated as a new platform, includes the mobile cellular, Internet of Things (IoT), ocean, satellites, Wireless Body Area Networks (WBANs), and yet-to-be-defined networks [3], [4].

WBANs arise as a viable option for e-healthcare as the global population increases and several severe illnesses, including emerging COVID-19, are testing healthcare systems [5]. In one case, a patient may obtain remote healthcare at home thanks to WBANs, eliminating the requirement to visit the hospital for periodic checkups [6], [7]. The WBANs are an exciting research domain that offers implanted and wearable Bio-Medical Sensor Nodes (BMSNs) technologies, communications, and platforms and is altering healthcare, quality of life, and physician-patient interactions [8], [9]. The adoption of WBAN has increased recently because of their flexibility and adaptability. These BMSNs can transmit biological information from the human body to a coordinator via wireless communication.

A BMSN should be small and low-power, capable of detecting medical signals such as Electrocardiograms (ECGs), Electroencephalograms (EEGs), pulse rates, blood flow, pressure, and temperature. The diversity in required data rates for various medical applications is evident. Parameters like ECGs, EEGs, pulse rates, blood flow, pressure, and temperature typically necessitate data rates below 10 kbps. In contrast, EMG demands no more than the data rate of 100 kbps. This study adopts the established IEEE 802.15.4 WBAN standard's appropriate data rate of 250 kbps for a patient monitoring system [10], [11], [12]. The coordinator then transmits the data to distant places to diagnostic and therapeutic reasons [9], [13]. As a result, the WBAN can establish a wireless communication network. WBANs employ heterogeneous-natured sensor nodes. These sensor nodes' traffic sources varied in energy consumption, computing, storage capacity, and data creation rate [14], [15]. The heterogeneous sensor nodes generate a range of medical data packets. Some medical data packets may sustain losses but must be transmitted on time, whilst others cannot tolerate multiple losses but must be supplied on time. Furthermore, although some medical information packets must be provided with no or minimal loss and within a certain period, others do not. Because of their intricacies, heterogeneous WBAN node activities are challenging to regulate [16], [17].

Wireless Local Area Networks (WLANs), Wireless Bluetooth, Wireless Personal Area Networks (WPANs), ZigBee, and other communication technologies are all integrated with WBANs. Typically, IEEE 802.15.6 and IEEE 802.15.4 are used by WBANs of communications at the Physical layers (PHY) and Media Access Control (MAC) [17], [18]. The newest member of the WPAN family, IEEE 802.15.6, is currently under development. IEEE 802.15.6 aims to supply a global standard for dependable, short-range wireless connection within the human body's reach. It can support up to 10 Mbps of data transfer for various applications [18], [11], [19]. Operating above the IEEE 802.15.4 MAC layer, ZigBee is a widely used industrial standard easily obtainable in completed form. Due to its low energy consumption and availability of several channels, IEEE 802.15.4 is highly recommended.

**Figure 1:**
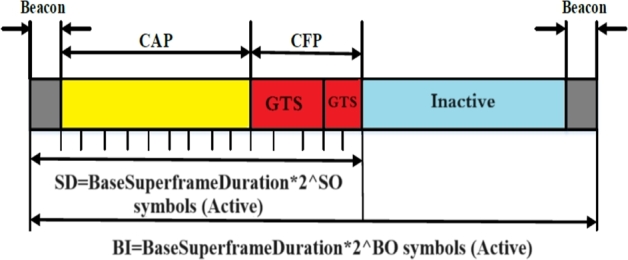
IEEE 802.15.4 beacon-mode frame format [25], [26].

IEEE 802.15.4 was established as a Low-Rate Wireless Personal Area Network (LR-WPAN) designed for tiny communication areas, lower data rates, real-time traffic and low energy consumption. Due to its unique characteristics, the IEEE 802.15.4 standard has emerged as the de facto protocol for WBANs despite not being initially designed for this specific application [20], [21]. MAC and PHY are both defined in the IEEE 802.15.4 standard. Non-beacon-enabled and beacon-enabled modes are the two operational modes specified by the standard. This study focuses on the coordinator to superframe structure primarily used for WBAN applications [22]. The beacon-enabled mode can be categorized into two primary portions, as indicated in Fig. 1: active and inactive periods. During the active phase, all nodes communicate with the coordinator to conserve energy while they enter a sleep state during the subsequent idle phase [23]. The IEEE 802.15.4 standard introduces two parameters, namely the Superframe Order (SO) and the Beacon Order (BO), to govern the superframe structure. Increasing the BO value extends the beacon interval (BI) duration. The active period of the beacon-enabled mode consists of two parts: the Contention-Free Period (CFP) and the Contention Access Period (CAP), also referred to as the Superframe Duration (SD), which are regulated by the SO parameter [24].

When utilizing the superframe structure, the SO and BO are initialized with default values that stay constant throughout the operation. However, superframe structure management is required for networks with unexpected traffic circumstances, which hurt network performance regarding channel utilization, packet buildup, and buffer overflow [27], [25]. The accumulating packets raise the likelihood of a collision even more. As a result, average energy usage increases for per-packet transmission and lowers network lifetime, but the average packet delivery ratio falls. Furthermore, the proper SO and BO adjustment for the superframe construction saves significant energy [28], [29]. However, it cannot attain a high packet delivery ratio because of an increased collision likelihood. BO and SO must be updated in every BI to ensure maximum throughput while using minimal energy. After each BI, the SO may be adjusted for an adaptive change in the superframe structure or active period.

As a result, to address the issues raised above, a dynamically adapting energy-efficient superframe structure scheme is expected. The study proposes a Takagi, Sugeno, and Kang (TSK) fuzzy logic controller to adjust the superframe structure for the WBAN-based IEEE 802.15.4 dynamic protocol in a star topology. This solution provides an appropriate solution for problems related to estimating information network traffic. Fuzzy logic instead of classical linear controllers or mathematical models is widely adopted in the IEEE 802.15.4 protocol because it gives an easy means of a specific conclusion imprecise, ambiguous, or vague input information [30], [31], [32]. In this study, a novel Dynamic Next Beacon Interval and Superframe Duration (DNBISD) scheme to adjust the synchronization process and time slot using the CAP due to traffic congestion and buffer overflow problems that occur with low, medium, or high traffic loads and also to enhance the Packet Delivery Ratio (PDR) for heterogeneous WBANs based on the TSK fuzzy model are proposed. Major contributions of DNBISD are:

• DNBISD adapts the synchronization process of the IEEE 802.15.4 standard according to traffic requirements. This improves the generated periodic beacon frames between sensor nodes and the coordinator.

• CAPs have been reduced, and their slot size increased according to each node's estimated information network traffic flow.

• The coordinator scrutinizes the next superframe structure by applying the TSK fuzzy model and fuzzy rules to calculate the packet received ratio and buffer ratio as input parameters.

• An adaptive solution enhances throughput, decreases loss packet, minimizes energy consumption and ensures high reliability. The simulation used Castalia-3.3 to show how effective the recommended approach is.

The paper is formatted in the following way. Section 2 describes the outline of IEEE 802.15.4. The related work is discussed in Section 3. Section 4 presents the proposed scheme. The simulation and results are shown in Section 5. Finally, Section 6 explains the concise summary of this research paper.

## 2 Outline of IEEE 802.15.4

**Figure 2:**
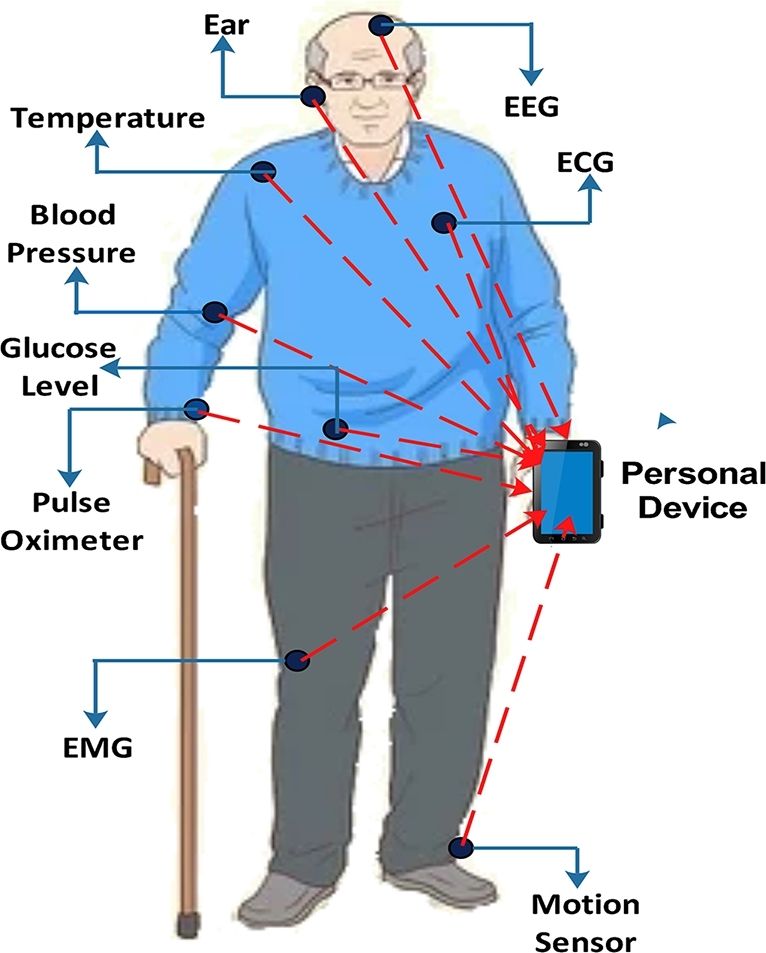
WBAN star topology.

An unresolved healthcare WBAN problem is the limitations of low-power coordinator and sensor nodes, particularly when the application demands frequent data transfer, which increases power consumption [33], [34]. This section offers the most significant WBAN standards to handle the limited coordinator and node resources used at the star topology, as shown in Fig. 2.

IEEE 802.15.4 allows low data speeds, prices, and energy consumption in WPANs. This protocol specifies the PHY and MAC layers while leaving the top levels to alternative approaches. IEEE 802.15.4 includes two modes: unslotted and slotted [35], [36]. The unslotted mode, called the non-beacon-enabled mode, involves nodes communicating dispersedly using unslotted-based CSMA/CA [37]. The slotted mode, which is beacon-enabled, divides time into equal-sized time slots. All devices in this mode should conform to a single-time superframe. The BI is the superframe's length that reflects the time between two beacon frame broadcasts. The coordinator periodically emits beacon frames to synchronize the associated devices and facilitate the connection of new devices to the network [25].

The BI has been split into two halves: an elective inactive period and an active period. To save energy, all devices—including the coordinator—enter a low-power mode when idle. Depending on the specification and setup, the device may reach an active state during the active period [38], [39]. The SD is the duration of the active period, which is categorized into 16 equal-sized time slots. The superframe time is split into two parts: CAP and CFP. Devices requiring dedicated bandwidth and utilizing TDMA can allocate CFP slots, also known as guaranteed time slots (GTS), with a maximum of seven slots. In the CAP, devices employ slotted CSMA/CA to contend for medium access, with a maximum of nine slots [40], [41].

In Fig. 1, a superframe structure is shown. The coordinator's BO and SO parameters control the beacon interval and superframe duration, respectively. These parameters are shown as functions of the beacon interval and superframe duration. The pair summarize the temporal properties of the superframe (SO, BO) [40].

**Figure 3:**
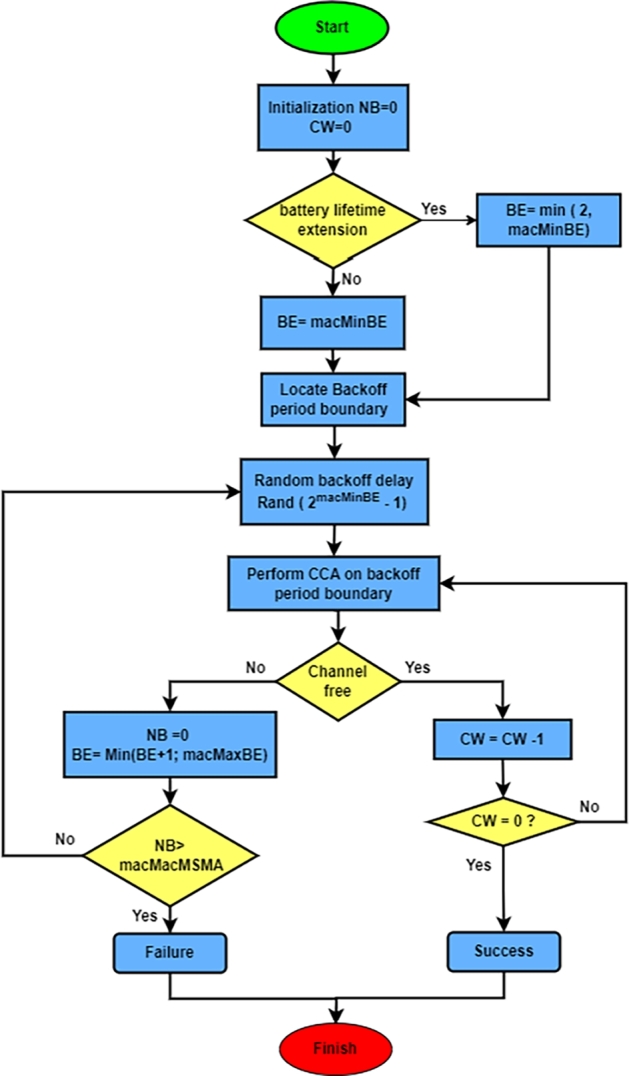
Data transmission flow diagram for IEEE 802.15.4 active period [45], [46].

Sensor nodes compete for the channel during active time by employing the slotted CSMA/CA algorithm seen in Fig. 3. Each sensor node monitors three transmit parameters: contention windows (CW), the numeral of backoff attempts (NB), and the backoff exponent (BE) [42], [41]. For a fresh transmission attempt, NB is set to zero. When the channel is determined to be busy or when a new transmission starts, the CW length parameter resets to 2. BE is a numeral of backoff slots before trying channel access that a device should wait for, with each slot representing 20 symbols. There are two kinds of backoff exponents: minimum backoff exponents (*macMinBE*) and maximal backoff exponents (*macMaxBE*). The macMinBE function is used to set the value of BE [43], [44], [45].

## 3 Related work

This section reviews prior research aiming to enhance IEEE 802.15.4 MAC efficiency by adjusting superframe elements. The current literature on the optimization of IEEE 802.15.4-based MACs may be separated into numerous techniques, and we will examine one of these approaches, superframe structure-based and heterogeneous traffic, in this study, which will be employed in our proposed study [45], [46]. A substantial amount of recent research has addressed the superframe structure method and low, medium, and high data traffic requirements for optimizing the execution of the MAC protocol based on IEEE 802.15.4 [18], [47], [48].

The researchers created the dynamic beacon interval and superframe adaptation algorithm (DBSAA) [49], which alters a DC based on BO and SO modifications. Furthermore, the coordinator node constantly modifies SO and BO depending on four parameters: superframe occupancy, number of received packets for the coordinator, collision ratio, and network node count. DBSAA is more DC adaptive, which results in energy savings, higher throughput, and reduced delay. DBSAA, on the other hand, supposes that all network sensor nodes use the same data rate. However, this algorithm needs to adjust SO and BO values correctly due to high energy consumption and is unsuitable for controlling medium and high data traffic.

The study by Rasouli et al. [50] suggested an Adaptive Duty Cycle Algorithm (ADCA) to solve energy consumption issues and challenges to beacon-based wireless sensor networks. The protocol improves the ability to estimate network traffic and model prediction and changes network DC. Coordination nodes collect network information, including queues and idle time on nodes. The coordinator also chooses and forecasts the next superframe order. However, this algorithm needs to correct the selection of the SO value due to high collisions and is unsuitable for controlling high-data traffic.

Another proposed protocol [46] uses MAC parameter tuning and DC-based on a Tele-Medicine Protocol (TMP). This protocol offers acceptable reliability and provides limited delay, making it suitable for patient monitoring applications. Delay-reliability factor, superframe duration, and traffic load are three parameters responsible for varying the DC, such as estimation of the network traffic, channel access, and probabilities of the collisions, which are the computation and functionality methods upon which this protocol is designed and based. However, this protocol is unsuitable for controlling medium and high data traffic, and the reduced active period increases the number of packets lost, and the PDR decreases.

The authors [10] present a new mathematical model for the BSN architecture that explains the location and connectivity of biosensor nodes, critical relay nodes, sink nodes, and network encoding relays. The model employs random linear network code (RLNC) and coordinate duty cycle (CDC) methods to enhance power efficiency in congested locations. The proposed technique minimizes power consumption; nevertheless, the suggested method is unsuitable for controlling high data traffic and is not used in conjunction with an actual BSN.

As used to the low-powered Internet of Medical Things (IoMT), the authors suggested the Content-based Dynamic Superframe Adaptation algorithm (CDSA) [36], which dynamically modifies the BO and SO in order to the BE of equilibrium the trade-off between the application's needs concerning network throughput, consumption of energy, communication delay, and packet delivery ratio. The CDSA approach does not need any modifications to IEEE 802.15.4. The coordinator employs a dynamic technique to control the BO and SO. The predicted SO is determined when the BI expires. When the network load is validated, the superframe structure is altered. When there is no network activity, the CDSA algorithm adjusts an SO and BO to default tuning to save energy. However, this algorithm has inconsistent traffic creation in various sensor devices and needs to be more suitable for controlling high data traffic.

The authors suggested an Auto-Management of Energy (AME) in IoT networks [51] for the IEEE 802.1.5.4 standard based on a WSN. A mathematical technique for estimating energy use was created to enable the coordinator to determine the battery's current power level. Based on the cluster tree network design, the suggested adaptive intervention compares the remaining energy and thresholds. The network is divided into subgroups with their own IEEE parameter (BO and SO). However, this algorithm does not consider other network factors, such as congestion-level traffic deadlines. PDR and throughput must be more suitable for controlling low and medium data traffic.

Previous works like AME, DBSAA, ADCA, TMP, CDC, and CDSA were assessed for their impact on power consumption, delay, throughput, latency, dependability, and overall network performance. However, under medium to high traffic loads, these studies reveal limitations for the coordinator. Issues include significant packet and energy loss during synchronization, leading to a low packet delivery ratio and buffer overflow. Additionally, problems arise when sensor nodes send data to the coordinator during fixed time slots using CAP, resulting in decreased throughput, higher network delay, prolonged node association duration, and increased energy consumption, ultimately degrading network performance.

Therefore, this study has filled the gap in the literature review by proposing a Dynamic Next Beacon Interval and Superframe Duration (DNBISD) scheme that can decrease loss packets, minimize energy consumption, increase reliability, and ensure enhanced throughput.

### 3.1 Fuzzy logic controlling

This study introduces a fuzzy logic-based controller instead of classical linear controllers or mathematical models because it gives a straightforward approach of a clear conclusion based on vague, imprecise, or ambiguous input information [30], [31], [32]. Fuzzy systems are extremely useful when dealing with a highly complex system with poorly understood behaviors and when an approximate but fast solution is required [52], [53], [54].

**Figure 4:**
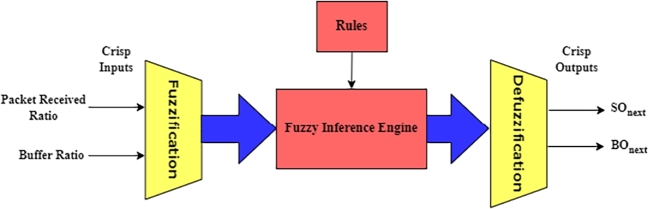
Structure Fuzzy Logic Control for proposed BOnext and SOnext [45], [46].

Fig. 4 illustrates the fundamental elements of a fuzzy system that uses rule-based inference engines, defuzzification, and fuzzification to process input language variables and produce output linguistic variables. The rules of a fuzzy logic system (FLS) are used to turn sharp inputs into sharp outputs. Defuzzification would result in the acquisition of crisp numbers. In a control system, for epitome, this number represents a control action, but in signal processing, it may represent a prognosis or a financial estimate [55], [32], [52].

Fuzzification: In a given universe, fuzzification transforms the space designated for system inputs (crisp inputs) into fuzzy sets. Every system input has a membership function assigned to it: Whereas $x_{i}$ denotes a unique input value, F represents a fuzzy set, and fuzzifier denotes a fuzzification operator, $F=fuzzifier\left(x_{i}\right)$ [56], [57]. Base Rule: A fuzzy rule can be described as a conditional statement such that, “If x is A, then y is B,” wherever A and B are linguistic values that are denoted by fuzzy sets that are specified on the universes X and Y, and x and y are linguistic variables. The “and” or “or” operators can combine the many parts of a fuzzy rule [53], [54]. The Inference Engine: The inference engine utilizes reasoning to generate conclusions based on knowledge and fuzzy inputs. All fuzzy rules will become active and merge at the same time. Defuzzification: Defuzzification is transforming the fuzzy output values of the inference mechanism into crisp ones [58], [56], [54].

Mamdani's [59] inference approach, with whose premise and the derived parameters were stated utilizing fuzzy sets, is utilized by many fuzzy systems. The Mamdani approach offers the theoretical foundations for fuzzy logic, and its fuzzy rules look as shown in Equation (1).

(1) $$If\;x_{1}\;is\;A_{1j}(m_{1j},\sigma_{1j})\;and\;x_{2}\;is\;A_{2j}(m_{2j},\sigma_{2j})\;...\;and\;\;x_{n}\;is\;A_{nj}(m_{nj},\sigma_{nj})\;Then\;y\;is\;B_{j}(m_{j},\sigma_{j})$$

As an output variable, it converts the if-then rule's consequence parameter into a fuzzy set. In contrast, $m_{nj}$ and $\sigma {}_{nj}$ represent a function of Gaussian membership of deviation and means for the nth dimension and the jth rule, respectively [53], [30]. TSK also presented the TSK fuzzy mode [60], a modified inference approach. The first two phases were identical to those in the pristine Mamdani model, fuzzing the inputs and making the process inference fuzzy. Additionally, rather than using fuzzy sets as the following parameters, such a type from the TSK fuzzy model creates a linear set for crisp inputs. The fuzzy rule standard of the TSK fuzzy model is illustrated in Equation (2).

(2) $$If\;x_{1}\;is\;A_{1j}(m_{1j},\sigma_{1j})\;and\;x_{2}\;is\;A_{2j}(m_{2j},\sigma_{2j})\;...\;and\;\;x_{n}\;is\;A_{nj}(m_{nj},\sigma_{nj})\;Then\;y\;=p_{0j}\;+p_{1j}x_{1}+\;...\;+p_{nj}x_{n}$$

In the TSK inference system, whereas $p_{nj}$ defines the degree of influence of each system input parameter, the defuzzification phase was unnecessary since the output of a rule is sharp [30], [31]. In contrast, the average weighted outputs for the crisp rule generated the model output. This process is shorter than the pristine centroid methodology's defuzzification. For a quantitative analysis of the whole system, the TSK fuzzy model outperforms the Mamdani technique [60], [32], [52].

Previously considered algorithms like AME, DBSAA, ADCA, TMP, CDC, and CDSA relied on traditional mathematics, rendering them unsuitable for WBAN amid unpredictable shifts in heterogeneous traffic load conditions. Consequently, this study advocates for DNBISD, leveraging the TSK fuzzy model for its robustness in handling variations and unexpected changes in heterogeneous traffic load conditions, making it well-suited for real-time decision-making.

## 4 Propose scheme

This section details the proposed design, the Dynamic Next Beacon interval and Superframe Duration scheme (DNBISD), to achieve high PDR for remote patient monitoring of biomedical applications based on IEEE 802.15.4 heterogeneous WBANs. Let us get started by specifying the underlying network assumptions first. The technique for calculating the factors that define network traffic is then described. Finally, we will go over the DNBISD operation.

**Figure 5:**
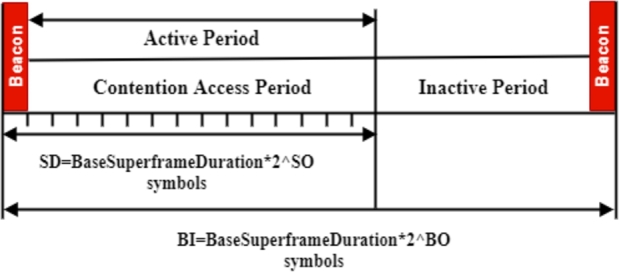
Design DNBISD Superframe Structure.

First, let us make some assumptions about the network mode of operation and configuration. Initially, the scheme is proposed for the network 802.15.4, which employs the topology of a star with the coordinator and nodes. Following that, we decided to use the coordinator to create the beacon period and the CAP portion in the synchronized and active periods, as shown in Fig. 5.

By replicating human decision-making, fuzzy logic may manage assessment issues with complicated variables and circumstances in the traffic flow of information-carrying packets. It gives a practical approach for the coordinator to make a judgment based on the language of the description to handle the input data in the manner of human operators. The DNBISD scheme's pseudocode algorithm is given in Algorithm 1.

**Algorithm 1:**
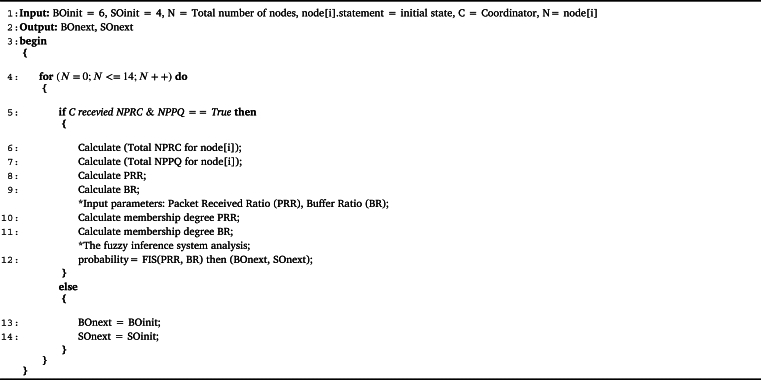
The DNBISD scheme.

**Figure 6:**
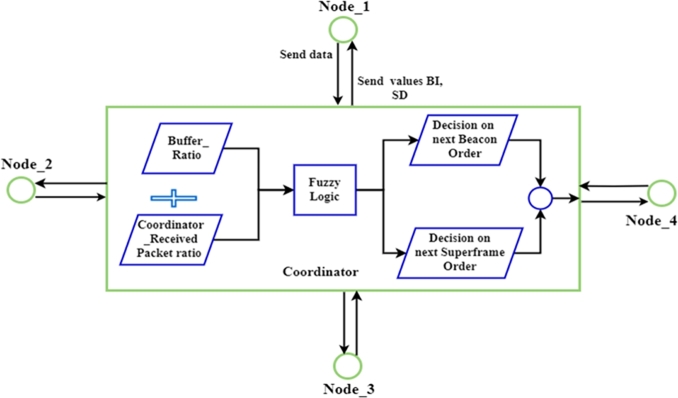
Design DNBISD scheme.

The proposed DNBISD scheme for WBAN employs fuzzy logic. It relies on two input parameters to calculate and estimate information network traffic from each node, such as Packet Received Ratio (PRR) and Buffer Ratio (BR) as input values for a coordinator. The coordinator calculates the output values for the next Beacon Order (nBO) and next Superframe Order (nSO) under various load situations after the CAP. Fig. 6 depicts the primary flow of the notion underlying our approach, which is characterized as follows:

• Packet Received Ratio (PRR): The packet received ratio variable indicates attribution for the Total Numeral of Received Packets to the coordinator (TNRP) to the Total Numeral of Sender Packets via nodes (TNSP), as shown in Equation (3).

(3) $$PRR=\frac{TNRP\;to\;the\;coordinator}{TNSP\;from\;nodes}$$

• Buffer Ratio (BR): The buffer ratio variable indicates the total number of hanging packets in the buffer of the sensor node to the total number of nodes that have packets hanging in the buffer. In other words, the buffer ratio variable indicates the Total Pending Buffer (TPB) to the Total Number to Nodes that have Packets Pending at the queue (TNNPP), as shown in Equation (4).

(4) $$BR=\frac{TPB}{TNNPP\;the\;queue}$$

In order to perform the fuzzification analysis at the final stage of the CAP, a coordinator converts input parameters for the PRR and BR into fuzzy linguistic variables. Equations illustrate their fuzzy sets, as shown in Equations (5) and (6).

(5) $$A_{1}(PRR)=\{\;X_{1}=”L”,\;X_{2}=”NL”,\;X_{3}=”MED”,\;X_{4}=”NH”,\;X_{5}=”H”\}$$

(6) $$A_{2}(BR)=\{\;X_{1}=”empty”,\;X_{2}=”medim\;”,\;X_{3}=”full”\}$$

So we see, the membership degree division for PRR is “L” low, “NL” nearlow, “MED” medium, “NH” nearhigh, and “H” high, but the membership degree division for BR is “empty”, “medium”, and “full”.

(7) $$\mu TRI\;(x)=\;\left\{ \begin{array}{cc} 0 & x⩽a_{1}\\ \frac{x-a_{1}}{b_{1}-a_{1}} & a_{1}⩽x⩽b_{1}\\ \frac{c_{1}-x}{c_{1}-b_{1}} & b_{1}⩽x⩽c_{1}\\ 0 & c_{1}⩽x \end{array}\right.$$

(8) $$\mu TRA\;(x)=\;\left\{ \begin{array}{cc} 0 & x⩽a_{2}\\ \frac{x-a_{2}}{b_{2}-a_{2}} & a_{2}⩽x⩽b_{2}\\ 1 & b_{2}⩽x⩽c_{2}\\ \frac{d_{2}-x}{d_{2}-c_{2}} & c_{2}⩽x⩽d_{2}\\ 0 & d_{2}⩽x \end{array}\right.$$

**Figure 7:**
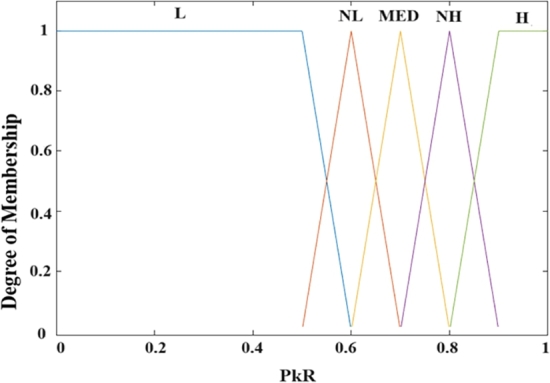
Membership function for Packet Received Ratio.

**Figure 8:**
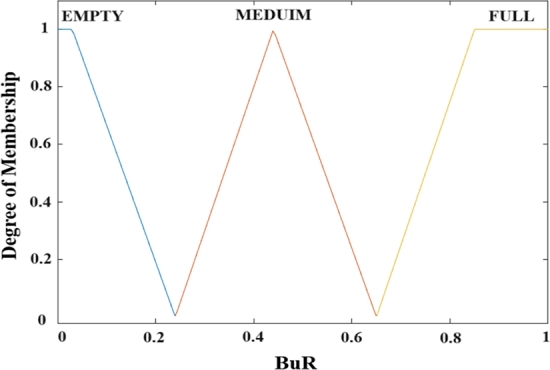
Membership function for Buffer Ratio.

The Triangular (TRI) function for membership is illustrated in Equation (7), and the Trapezoidal (TRA) function of membership illustrated in Equation (8) are the most popular functions of membership for fuzzy inference systems. The membership degree in their corresponding functions of membership, which determine a change of dynamic to the related fuzzy linguistic variable, is represented by TRA(x) and TRI(x). The mapping values corresponding to the triangle's three vertices on the X-axis are $a_{1}$, $b_{1}$, and $c_{1}$. The mapping values corresponding to the trapezoid's four vertices on the X-axis are $a_{2}$, $b_{2}$, $c_{2}$, and $d_{2}$. In universal, a triangular function of membership was utilized to boundary variables, while a membership of triangular function was used at variables that are intermediate. Moreover, we may derive the general function of membership for the related parameter by adding the function of membership for every linguistic variable at the set of fuzzy variables of the subordinate parameter. These fuzzy input parameter functions of membership are defined using some of our experimental experiences, as shown in Figure 7, Figure 8.

Fig. 7 depicts the membership function of the packet received ratio degree. These membership functions are employed throughout the fuzzification process. The five membership functions for “L”, “NL”, “MED”, “NH”, and “H” correspond to the fuzzy elements (“L”, “NL”, “MED”, “NH”, and “H”) contained in the PRR fuzzy set. They transform the current crisp PRR to the appropriate degree of membership set specified as the variable fuzzy. For example, if PRR is 0.47, corresponding of the “L” function, we may get the degree of membership as 0.58; for the “NL” function, we may get the degree of membership as 0.64; and for the “MED” function, we may get the degree of membership as 0.74; for the “NH” function, we may get the degree of membership as 0.82; and for the “H” function, we may get the degree of membership as 0.82. Consequently, we may get the appropriate PRR membership set (0.47, 0.58, 0.64, 0.74, 0.82), the PRR fuzzy variable, and engage in the following fuzzy logic analysis. The same thing is true for Fig. 8, which depicts the membership function of the buffer ratio degree. The three membership functions for “empty,” “medium,” and “full” correspond to the BR degree fuzzy set's fuzzy elements (“empty,” “medium,” and “full”). As the fuzzy variable, they transform a current crisp BR degree into the appropriate degree of membership set.

Figure 7, Figure 8 demonstrate the segmentation function of membership for whole input fuzzy linguistic variables. The fuzzy inference system will convert the pristine, crisp values to fuzzy linguistic variables. Furthermore, as demonstrated in Equation (9), we may develop IF-THEN rules based on the technique of the TSK system of deductive reasoning. The DNBISD scheme's outcomes IF-THEN rules table is shown in Table 1.

(9) $$If\;(PRR_{j}\;is\;MF)\;and\;(BR_{j}\;is\;MF)\;then(nSO_{j}\;=\;p_{0j}+\;p_{1j}(PRR)\;+\;p_{2j}(BR))\;and(nBO_{j}\;(X)=\;p_{0j}+\;p_{1j}(PRR)\;+\;p_{2j}(BR))$$

**Table 1:** Fuzzy Inference Rules.

List	PRR	BR	nSO	nBO
1	L	EMPTY	2	4
2	NL	EMPTY	3	4
3	MED	EMPTY	5	6
4	NH	EMPTY	6	6
5	H	EMPTY	7	7
6	L	MEDUIM	3	4
7	NL	MEDUIM	4	5
8	MED	MEDUIM	5	6
9	NH	MEDUIM	6	7
10	H	MEDUIM	7	8
11	L	FULL	5	5
12	NL	FULL	5	6
13	MED	FULL	5	7
14	NH	FULL	5	8
15	H	FULL	5	9

Using the input function membership mentioned above, the Fuzzy Inference System (FIS) transforms the pristine crisp input variables to match the linguistics of fuzzy variables. In the fuzzy logic discipline, the most frequent way to model human thinking is to construct language formulations incorporating the commonly used IF premise, THEN conclusion. Furthermore, for the IF-THEN rule-based representation of knowledge constructed from natural language descriptions and models, the premise was a decision condition indicated via the linguistic of fuzzy variables at the set of fuzzy input parameters, and its conclusion can be considered an output of the fuzzy variable. It denotes that the fuzzy engine computes output variable values by encapsulating expert assessment system knowledge. Table 1 displays the actual data from earlier research as well as the rules of fuzzy. However, the TSK technique was used in a fuzzy inference system based on a one-order Sugeno system. Because two input parameters are utilized, the first has five levels, and the second has three levels for the two output parameters. The output (consequent) system has taken the form of constant equations rather than a fuzzy set, as shown in Equations (10) and (11).

(10) $$nSO_{j}\;(X)=\;p_{0j}+\;p_{1j}(PRR)\;+\;p_{2j}(BR)$$

(11) $$nBO_{j}\;(X)=\;p_{0j}+\;p_{1j}(PRR)\;+\;p_{2j}(BR)$$

The definition of a weighted average system is the two final outputs of all rules, as shown in Equations (12) and (13).

(12) $$\overset{ˆ}{Finaloutput_{1}}=\frac{\sum_{j=1}^{m}nSO_{j}\;w_{j}1}{\sum_{i=1}^{m}w_{j}1}$$

(13) $$\overset{ˆ}{Finaloutput_{2}}=\frac{\sum_{j=1}^{m}nBO_{j}\;w_{j}2}{\sum_{i=1}^{m}w_{j}2}$$

Equations (14) and (15) show that the membership function in the jth rule is weighted by the firing strength linguistic variable of the fuzzy input parameter, wherein m is the number of fuzzy rules.

(14) $$w_{j}1=\;MIN\;(\mu (PRR),\;\mu (BR))$$

(15) $$w_{j}2=\;MIN\;(\mu (PRR),\;\mu (BR))$$

To make the computation more accessible, we define $T_{j}1$ and $T_{j}2$, as shown in Equations (16) and (17). Then, we must characterize the final result as shown in Equations (18) and (19).

(16) $$T_{j}1=\frac{w_{j}1}{\sum_{j=1}^{m}w_{j}1}$$

(17) $$T_{j}2=\frac{w_{j}2}{\sum_{j=1}^{m}w_{j}2}$$

(18) $$\overset{ˆ}{Finaloutput_{1}}=\sum_{j=1}^{m}T_{j}1⁎nSO_{j}(X)≃\;\sum_{j=1}^{m}T_{j}1(p_{0}^{j}\;+(p_{1}^{j}A_{1}(PRR)\;+(p_{2}^{j}A_{2}(BR))$$

(19) $$\overset{ˆ}{Finaloutput_{2}}=\sum_{j=1}^{m}T_{j}1⁎nBO_{j}(X)≃\;\sum_{j=1}^{m}T_{j}1(p_{0}^{j}\;+(p_{1}^{j}A_{1}(PRR)\;+(p_{2}^{j}A_{2}(BR))$$

To compute the proposed DNBISD Scheme, such as SD next, BI next, Sleep Period next, and Duty Cycle next (DCn) as shown in Equations (20), (21), (22) and (23).

(20) $$SD_{next}=aBaseSuperframeDuration⁎2^{nSO_{j}}symbols\;0⩽\;nSO⩽\;nBO⩽\;14$$

(21) $$BI_{next}=aBaseSuperframeDuration⁎2^{nBO_{j}}symbols\;0⩽\;nBO⩽\;14$$

(22) $$SleepPeriod_{next}\;=BI_{next}\;-\;SD_{next}$$

(23) $$DutyCycle_{next}\;=\frac{SD}{BI}=\frac{2^{nSO_{j}}}{2^{nBO_{j}}}=2^{nSO_{j}-nBO_{j}}\;⁎100$$

## 5 Simulations and results

Our work utilizes the CASTALIA 3.3 simulator, constructed on the Omnet++ platform, and offers numerous advantages [61], [62]. It is a complete and objective modular network testbed built in C++, aiming to be the most realistic WBAN simulator accessible. This framework may be customized for numerous uses and enables discrete-event network simulation and object-oriented modularity, making it a multidisciplinary tool [63], [19]. Table 2 outlines the simulation parameters used in our study. Our network consists of 14 sensor nodes and one body coordinator arranged in the topology of a star, as illustrated in Fig. 1. At the start of the superframe, the coordinator broadcasts beacon signals, followed by an acknowledgment when the packet is received. Nodes fight for the channel during the active period, utilizing slotted CSMA/CA, while they sleep during the inactive period to preserve energy. We estimate various duty cycle values based on updated rates of IEEE 802.15.4 parameters (BO, SO). The CFP is zero, indicating that the CAP covers the full active time. The simulation results are more realistic since they consider the amount of energy used.

**Table 2:** Setting the default MAC and Physical transmission parameters.

Parameters	Value	Parameters	Value
Number of nodes	14	batteryLifeExtention	False
Coordinator number	1	aUnitBackoffPeriod	20 symbols
Simulation Time	**Table:** - 1: (50-400) s - 2: 500 s	aBaseSlotDuration	60 symbols
Initial energy	18720 J	aNumSuperframeSlots	16
MAC Layer		Physical Layer	
MacMinBE	1	Frequency band	2.4 GHz
MacMaxBE	5	Channel mode	**Table:** Long Shadowing Wireless Model
BO default	5	Bandwidth	20 MHz
SO default	4	CCAthreshold	-95 dBm
MacMaxCSMABackoff	4		
Buffer size	32		
MacFrameRetries	2		

With wearable or implanted monitoring devices, WBAN treats chronic illnesses like diabetes and hypertension and saves lives by monitoring physiological parameters and anticipating adverse circumstances. Implantable sensors cannot measure many essential signals since they need more time to survive for many months or years without assistance. On the other hand, wearable sensors can measure the most critical signals, such as heart rate monitor, temperature, oxygen saturation (SpO2), ECG, accelerometer, EEG, gyroscope, EMG, pulse oximeter, barometer, and blood pressure (BP) [17], [11], [12]. Our simulation scenario is related to wearable medical applications for patient monitoring in which patients are given limited sensor nodes.

After conducting six repeated simulations for each scenario, readings were acquired. The average reading, along with a 95% Confidence Interval (CI), was then calculated and documented. Therefore, we discuss the outcomes in two situations: The first scenario involves the production of sensor nodes from 2 to 14, respectively, during a fixed period of 250 s, with three levels of data traffic: low, medium, and high. Data traffic production rates of 4 packets per second are regarded as low, 15 packets per second as medium, and 30 packets per second as high. In the second scenario, we generate different times from 50 to 400 s for static 14 sensor nodes and a data rate of 25 pps.

In the first scenario, we assess the effectiveness of the suggested DNBISD scheme and the default IEEE 802.15.4 scheme. We compare the AME [51], CDSA [36], and default IEEE 802.15.4 schemes in the second scenario with the proposed DNBISD scheme. It comprises the average Packet Loss Ratio (PLR), throughput, PDR, and the coordinator's energy consumption.

**Figure 9:**
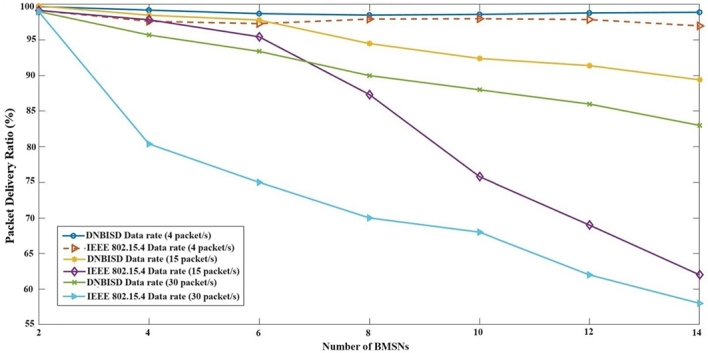
Packet Delivery Ratio for number of BMSNs.

Fig. 9 compares the PDR values for the proposed DNBISD and the default IEEE 802.15.4 schemes. It is observed that DNBISD achieves a 94.8% higher PDR for low, medium, and high data rates. This improved PDR can be attributed to the proper selection of MAC parameters, such as *macCSMABackoff* and *macFrameRetries*. These enhance reliability and the optimal next superframe order and next beacon order selection strategies. When the numeral of nodes increases and the data rate value is low, the PDR improvement ratio between DNBISD and the IEEE 802.15.4 are 1% for the proposed method, with the small data rate being the main factor. In the case of medium data rates, the improvement ratio is 11% for the proposed method. It is also worth noting that the proposed DNBISD method shows a 17% improvement ratio for high data rates compared to the IEEE 802.15.4 scheme. This improvement is due to the dynamic allocation of superframe order and beacon order for data traffic flow between networks in the DNBISD method, as opposed to the static scheme in IEEE 802.15.4, which contains a fixed CAP, which leads to a decline in dependability and an increase in collisions.

**Figure 10:**
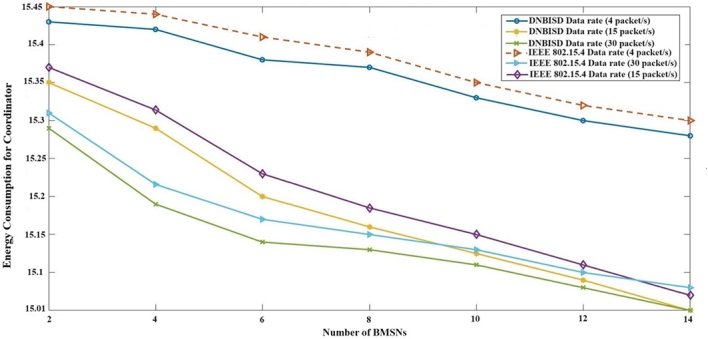
Energy Consumption to Coordinator for number of BMSNs.

Fig. 10 presents the average energy consumption to coordinator usage, revealing that the DNBISD scheme boasts lower energy consumption. The DNBISD scheme employs fuzzy rules within the coordinator to select the optimal SO and BO for the next period. As the number of nodes rises and the data rate fluctuates between low, medium, and high, an energy consumption improvement ratio between DNBISD and IEEE 802.15.4 varies, provided that it does not surpass an energy consumption of 0.02%. This improvement is due to the effective utilization of the energy ratio, which dynamically allocates the superframe and beacon orders based on fuzzy principles. In contrast, IEEE 802.15.4 networks experience idle listening initially, followed by transmission failure and high contention, causing energy waste.

**Figure 11:**
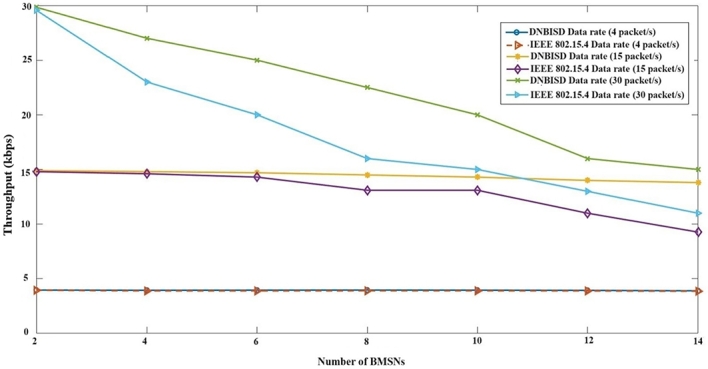
Throughput for number of BMSNs.

In Fig. 11, we can see how a coordinator impacts the network's overall throughput. The average throughput is the number of data packets a coordinator receives within a given time frame. According to our findings, the DNBISD scheme produces higher throughput than the IEEE 802.15.4 protocol when data rates vary from low to high and sensor node numbers increase. Our solution employs fuzzy rules that consider inputs such as the average numeral of received packets via a coordinator and the buffer rate in all nodes to predict the ideal values SO and BO for the next period, thereby increasing throughput. The DNBISD and IEEE 802.15.4 throughput performances are similar when low data rates are utilized. However, as data rates increase from medium to high and sensor node numbers rise, the differences between the two charts become more pronounced. As a result, data packet collisions consume more resources than they produce valuable data.

**Figure 12:**
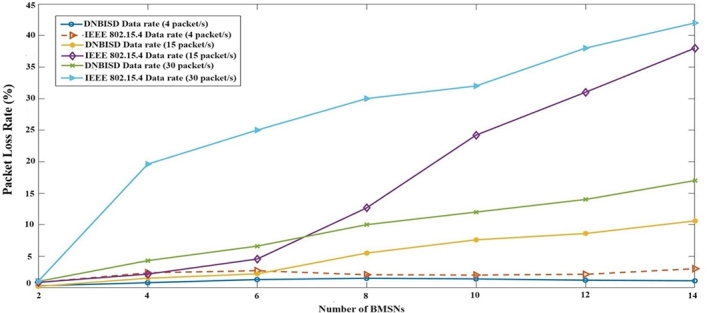
Packet loss Ratio for number of BMSNs.

Fig. 12 compares the PLR for the proposed DNBISD and default IEEE 802.15.4 schemes with different data rates and sensor nodes. The proposed DNBISD achieves a reduced packet loss rate of 5.17% compared to IEEE 802.15.4 at low, medium, and high data rates. An improved PLR is due to using the right SO and BO, which enhances reliability and reduces packet loss. The lost packet ratio for IEEE 802.15.4 and DNBISD is only 1% when the numeral of nodes increases and a data rate value is low, whereas the lost packet ratio for IEEE 802.15.4 is 11% for medium data rates from DNBISD. Additionally, the IEEE 802.15.4 scheme has a 17% higher rate of missed packets than the proposed DNBISD scheme at high data rates. Because IEEE 802.15.4 employs a static strategy with a fixed CAP, there are more collisions and fewer loss packets. At the same time, DNBISD dynamically allocates superframe order and beacon order for data traffic flow across networks, which enhances the loss packets.

**Figure 13:**
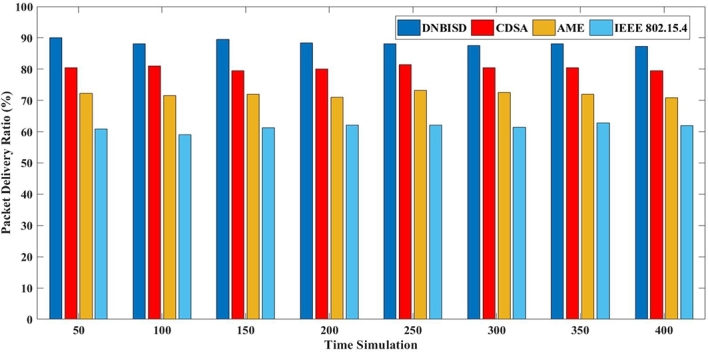
Packet Delivery Ratio for different simulation times.

**Figure 14:**
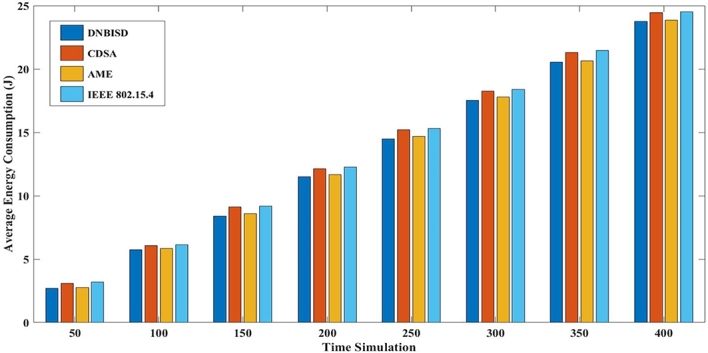
Average Energy Consumption for different simulation times.

**Figure 15:**
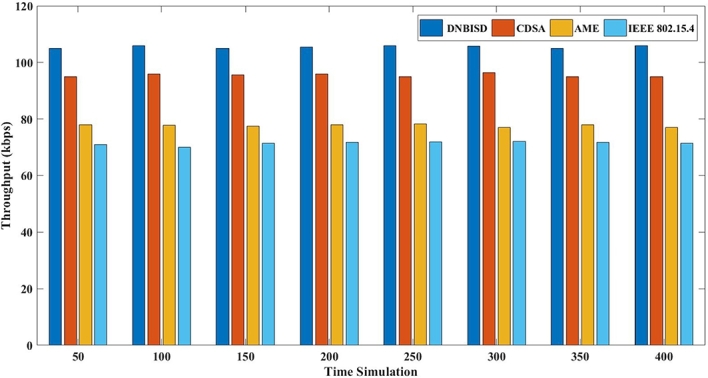
Throughput for different simulation times.

**Figure 16:**
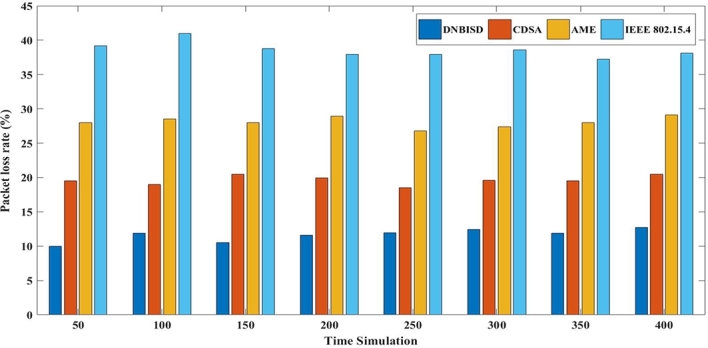
Packet loss Ratio for different simulation times.

The second set of trials included different simulation times from 50 to 400 s for 14 sensor nodes and a data rate of 25 pps. Figure 13, Figure 14, Figure 15, Figure 16 display the comparison results of PDR, average energy consumption for the coordinator, average throughput, and PLR to AME, CDSA, IEEE 802.15.4 and the proposed DNBISD schemes.

Fig. 13 illustrates the PDR for different superframe schemes' network dependability, which shows varying levels of dependability. The network's effective PDR may decrease if the synchronization and active period are not regulated to adapt the appropriate number of packets in the channel. While other schemes like AME and CDSA wait for the threshold value to be overridden before adjusting the superframe structure, the DNBISD scheme accurately predicts the changing network conditions. In contrast, IEEE 802.15.4 relies on a static amount for the superframe structure. At the same time, the systems mentioned above depend on metrics like collision rate, received frames, latency, and energy consumption limits to activate the superframe structure in the coordinator. Due to the constant data rate, the network endures longer simulation durations. As a result, DNBISD achieves approximately 8% to 26% more dependability than similar schemes.

Fig. 14 displays the coordinator network's average energy consumption for various schemes, including DNBISD, AME, CDSA, and IEEE 802.15.4, so channel traffic increases over time. DNBISD adjusts the dynamic BO and SO settings appropriately to progressively higher energy consumption with times, while AME and CDSA calculate the duty cycle of the superframe structure in the coordinator various factors such as collision rate, correctly received frames, energy consumption ceilings, and latency. In contrast, IEEE 802.15.4-based networks experience idle listening, high contention, transmission failure, and waste energy, leading to power depletion and network synchronization issues. The proposed DNBISD scheme is unique in its ability to effectively understand channel congestion, compared to the subpar measurements and predetermined thresholds used by AME, CDSA, and IEEE 802.15.4 systems. As a result, the DNBISD scheme has the lowest average energy consumption for the coordinator.

Fig. 15 displays the linearly static network throughput for the proposed DNBISD scheme due to using a fixed data rate for the simulation network's sensor nodes. However, the active time and synchronization are not controlled to adapt the appropriate amount to packets at the channel. In that case, the effective throughput of the network may decrease as the data rate increases. The DNBISD system properly forecasts the rise in channel traffic, while AME and CDSA wait until the threshold value is surpassed before altering the superframe parameters. According to parameters such as buffer rate and the quantity of correctly received data, the DNBISD system's throughput triggers properly in the superframe structure at the coordinator. Meanwhile, the superframe structure for IEEE 802.15.4 depends on a constant value. As a result, DNBISD offers 10% to 34% greater throughput than comparable systems.

Fig. 16 illustrates the PLR for various superframe methods depicting different loss amounts. The effective PLR of the network may increase if the active time and synchronization are not controlled to provide the appropriate amount of packets in the channel. However, the DNBISD system anticipates changing network conditions correctly, unlike other schemes such as AME and CDSA that wait for the threshold value to be surpassed before updating the superframe structure. On the other hand, the superframe structure for IEEE 802.15.4 is a fixed value. To activate the superframe structure at the coordinator, the systems mentioned above rely on criteria such as collision rate, received frames, latency, and energy consumption limits. Due to the consistent data flow, the network can endure prolonged simulation times, resulting in lesser data loss for the DNBISD scheme than comparable methods.

Both sets of simulation results show that the packet delivery ratio of the proposed DNBISD system is significantly higher at low and high data rates than that of current approaches. Consequently, the data-dropping ratio was lower, leading to an increase in average throughput. By utilizing dynamic Sugeno fuzzy logic to control the selection for the best output for the next superframe order (nSO) and beacon order (nBO) based on input parameters such as the packet received ratio and buffer ratio, the DNBISD scheme was able to reduce the packet loss ratio and average energy consumption significantly.

## 6 Conclusion

This study proposes the Dynamic Next Beacon Interval and Superframe Duration (DNBISD) technique to improve the dependability of the superframe structure in IEEE 802.15.4 WBAN and controllers utilizing the TSK fuzzy model. The main benefit of this control strategy is the coordinator's ability to dynamically build a superframe to manage the heterogeneous traffic load requirements of requesting nodes. If the available limit is exceeded during synchronized and active periods, the requested nodes might not be served, or BI and CAP might continue underutilizing. The fuzzy logic controller analyzes the packet received and buffer ratio to modify the coordinator's commands for the subsequent beacons and superframes. The suggested system, however, enables the coordinator to modify its beacon interval (BI) and contention access period (CAP) in response to various data demands from sensor nodes. The simulation results for the two scenarios demonstrated that when the data rate was low, medium, or high in the first scenario, the proposed scheme improved the packet delivery ratio and throughput, decreased the coordinator's average energy consumption, and reduced the packet loss ratio, as compared to IEEE 802.15.4. Additionally, the proposed method's second scenario can outperform AME, CDSA, and IEEE 802.15.4 regarding relevantly more excellent packet delivery ratio and throughput with lower average energy consumption in the coordinator and packet loss ratio. Future work will include implementing DNBISD on sophisticated testbed configurations. Additionally, we want to examine dynamic slot distributions to an already-existing transmission plan for MAC behaviors based on IEEE 802.15.4e. The fog-assisted network is also set up using various data rates for patient vitals.

## CRediT authorship contribution statement

**Abdulwadood Alawadhi:** Writing – original draft, Visualization, Validation, Supervision, Software, Resources, Project administration, Methodology, Investigation, Formal analysis, Data curation, Conceptualization. **Abdullah Almogahed:** Writing – review & editing, Visualization, Validation, Software, Methodology, Investigation, Formal analysis, Data curation, Conceptualization. **Fathey Mohammed:** Writing – review & editing, Validation, Supervision, Resources, Project administration, Investigation, Conceptualization. **Bakr Ba-Quttayyan:** Validation, Investigation, Formal analysis, Data curation, Conceptualization. **Adnan Hussein:** Visualization, Investigation, Formal analysis, Data curation, Conceptualization.

## Declaration of Competing Interest

The authors declare that they have no known competing financial interests or personal relationships that could have appeared to influence the work reported in this paper.

## Data Availability

Data included in article/supp. material/referenced in article.
